# Web-Based Health Information Technology: Access Among Latinos Varies by Subgroup Affiliation

**DOI:** 10.2196/10389

**Published:** 2019-04-16

**Authors:** Mariaelena Gonzalez, Ashley Sanders-Jackson, Tashelle Wright

**Affiliations:** 1 Department of Public Health University of California - Merced Merced, CA United States; 2 Health Science Research Institute University of California - Merced Merced, CA United States; 3 Department of Advertising and Public Relations College of Communication Arts and Sciences Michigan State University East Lansing, MI United States

**Keywords:** information seeking behavior, health information technology, Hispanic Americans, minority health

## Abstract

**Background:**

There are significant health technology gaps between Latinos and non-Hispanic whites and between first- and second-generation Latinos.

**Objective:**

This study aimed to examine disparities in Web-based health information–seeking behavior (HISB) and patient portal use among Latinos, taking into account nativity and subethnic affiliation.

**Methods:**

We analyzed US-born, non-Hispanic whites and Latinos adults (N=49,259) and adult internet users (N=36,214) in the 2015 to 2016 National Health Interview Survey using a binary logistic regression controlling for individual difference level variables. Outcomes were internet use, HISB (health information-seeking online and using a chat group for health information), and patient portal use (using a computer to schedule an appointment, filling a prescription, and communicating with a provider).

**Results:**

We found that US-born Mexicans (odds ratio [OR] 0.81, 95% CI 0.66-0.99), foreign-born Mexicans (OR 0.35, 95% CI 0.29-0.42), foreign-born Puerto Ricans (OR 0.62, 95% CI 0.44-0.87), foreign-born Central and South Americans (OR 0.42, 95% CI 0.33-0.53), and foreign-born other Latinos (OR 0.34, 95% CI 0.24-0.49) had lower odds of using the internet than US-born non-Hispanic whites. The relationship between subgroup affiliation and Web-based HISB varied by type of technology. US-born Mexicans (OR 0.77, 95% CI 0.66-0.9), foreign-born Mexicans (OR 0.51, 95% CI 0.43-0.61), foreign-born Central and South Americans (OR 0.53, 95% CI 0.43-0.64), and foreign-born other Latinos (OR 0.56, 95% CI 0.4-0.79) had lower odds of looking up health information online than US-born non-Hispanic whites. Controlling for age, sex, education, income to federal poverty level, and region, foreign-born Central and South Americans (OR 0.61, 95% CI 0.41-0.92) and foreign-born other Latinos (OR 0.26, 95% CI 0.1-0.68) had lower odds of filling a prescription using a computer than US-born non-Hispanic whites. Foreign-born Mexicans (OR 0.51, 95% CI 0.36-0.72) and foreign-born Central and South Americans (OR 0.7, 95% CI 0.5-0.99) have lower odds of emailing a health care provider than US-born non-Hispanic whites. Posthoc analyses were conducted among Mexican-Americans to see if age was significant in predicting Web-based HISB or other patient portal use. We found individuals aged 18 to 30 years had higher odds of using the internet (OR 3.46, 95% CI 2.61-4.59) and lower odds of looking up health information online (OR 0.75, 95% CI 0.58-0.96). A posthoc analysis was conducted among Mexican-Americans to see if nativity predicted Web-based HISB and patient portal use. We found that US-born individuals had higher odds (OR 52.9, 95% CI 1.2-1.93) of looking up health information online compared with foreign-born individuals.

**Conclusions:**

We found Latino subgroups do not use health information channels equally, and attempts to target Latinos should take ethnicity and nativity into account.

## Introduction

### Background

The use of Web-based health information technology (HIT) is spreading [1]. For example, encouraged by the Health Information Technology for Economic and Clinical Health Act, in 2012, at least half of all health care providers had adopted the use of patient portals [2]. Patient portals have been shown to have a wide variety of benefits, including the management of chronic disease; however, Latinos are also less likely to adopt patient portals [3,4]. Previous research has suggested disparities in internet access, online health information–seeking behavior (HISB) between non-Hispanic (NH) whites and Latinos, and between US- and foreign-born Latinos [5-11]. With the increasing reliance on patient portals [4], it is important to understand where disparities in specific types of online HISB and patient portal use occur for Latinos at the national level.

Although Latinos have historically lagged behind NH whites in access to the internet, there have been significant gains in internet access among Latinos between 2012 and 2015, particularly among Spanish speakers [8]. Despite this, only 46% of Hispanics access the internet through broadband, and a 2017 Pew Research Report stated that 22% of Hispanics did not have broadband but owned a smartphone [8,12]. These differential patterns of access and device use are disparities, and disparities in the use of Web-based HIT, such as patient portals, should also be investigated.

There are significant information knowledge gaps between Latinos and NH whites when it comes to Web-based patient portals [4,13-15]. In a systematic review of patient portal literature, although patient portals may improve outcomes, particularly in conjunction with a clinical intervention, for some patients with chronic conditions, racial and ethnic minorities may not receive the same benefit as whites [15]. Another systematic review found that ethnicity can play a role in lack of adoption of patient portals [4]. Indeed, in a California sample of elderly adults, Latinos were less likely to use patient portals than NH whites, although Latinos were not broken down into subgroups in this analysis [16]. Latinos also have lower rates of activating and logging into patient portals than NH whites [17,18].

Although some state- and local-level examinations of online HISB and the use of patient portals exist [9,19-22], and several national level studies [1,11], these studies do not control for other factors such as Latino subethnicity or nativity that might be important cultural variables that could contribute to or mitigate disparities.

When examining Latinos in the United States, nativity and ethnicity are important determinants of health and media use. Although Latinos are the largest ethnic group in the United States [23], there are significant demographic differences between US- and foreign-born individuals. On average, US-born Latinos, who make up over 60% of the Latino population in the United States, are younger and consume more English-language media [8,24]. US-born Latinos engage in more online HISB and have higher levels of confidence in their ability to fill out Web-based forms than foreign-born Latinos [9]. Although researchers commonly lump Latino subethnicities together, there are significant health and behavioral differences within and between the various subethnicities [25-28]. For example, research suggests that Puerto Ricans are more likely to be current smokers and to have ever smoked compared with Dominicans, Colombians, and Ecuadorian individuals [27]. Puerto Ricans also reportedly have higher rates of depression and suicide attempts compared with Mexican and Cuban Americans [26], Cuban Americans have higher systolic blood pressure and higher cholesterol than both Mexican Americans and Puerto Ricans [25], and perceptions of cancer risk factors vary across Latino ethnic subgroups [28]. A study in 2007, which examined internet use among Latinos by subethnicity, found that in the United States, internet use varies among ethnic groups, with South American origin Latinos having a higher than average rate of internet use and Mexicans and Central Americans having lower rates of internet use [29], which indicates that ethnicity is an important factor when examining internet-related behavior among Latinos. However, we did not find other studies on Latino internet use that disaggregate Latinos in this way. Due to the US or foreign-born (nativity) and ethnic differences among Latinos, it is important to take into account nativity and ethnicity, if possible, when examining disparities between NH whites and Latinos.

### Objective

The purpose of this study was to examine disparities in online HISB and patient portal use among NH whites, US-born Latinos, and foreign-born Latinos, taking into account subethnic affiliation and disaggregating subethnicities or regional groupings when possible. By understanding where disparities exist, we can effectively target interventions to increase online health literacy within the largest linguistically and culturally sensitive ethnic group in the United States.

## Methods

### Data

We combined 2 years (2015 and 2016) of the adult sample file of the National Health Interview Survey (NHIS), which is a nationally representative sample of the adult US civilian population. We examined all US-born whites and Latinos (N=49,251) and only those individuals who reported using the internet (N=36,214).

### Primary Outcomes

Respondents were asked if in the last 12 months they had used the internet or used email. In addition, they were asked if in the last year they had engaged in online HISB and used a computer to (1) “[l]ook up health information on the Internet,” [30] or (2) “[u]se online chat groups to learn about health topics” [30]. They were also asked if they had used patient portals by using a computer to (3) “[f]ill a prescription,” [30] (4) “[s]chedule an appointment with a health care provider,” [30] or (5) “[c]ommunicate with a health care provider by email” [30]. Each of these binary items was separately used as a primary outcome in our analyses. If respondents reported using the internet, email, engaging in online HISB or online patient portals in the last 12 months, they were classified as having used the internet in the last 12 months.

### Latino Subgroup Affiliation by Nativity

There are differences between US- and foreign-born Hispanics with regard to the median age and use of Spanish or English within the population [9,24,31,32]. There are differences between Hispanic subgroups and region, which may play a role in health care access [25-28]. Furthermore, different countries of origin may be associated with cultural differences that impact health access. In addition, studies have shown significant behavioral and health differences among various Hispanic ethnic groups, including around internet use [29,31-33]. Although combining 2 years of the NHIS did not yield a large enough sample to disaggregate all Latino ethnic subgroups in our model, we were able to obtain a large enough sample to divide Hispanics into the following subgroups: Mexican and Mexican-Americans (Mexicans), Puerto Ricans, Central and South American, and other Latino (Cuban, Dominican, Other Latin American affiliation). Although some subgroups we used contain large regions or disparate groups, we combined these groups based on Centers for Disease Control and Prevention’s classifications and because of sample size issues. Individuals who identified as *other Spanish* or *multiple Hispanic* were excluded as we could not specifically identify their ethnic or regional origin. We further divided each of the 4 Latino subgroups into US- and foreign-born individuals. Our comparison group for all Latino categories was US-born NH whites, as it is possible that foreign-born NH whites have different health outcomes and media use patterns than US-born individuals.

### Other Individual-Level Variables

We controlled for age (18-30 years, 31-54 years, and 55 years and above), gender (male, female), education (below a high school education, completed high school or general education development, some college, and completed a bachelor’s or higher), poverty level (0%-99% of the federal poverty level [FPL], 100%-199% of the FPL, and 200% or greater of the FPL), marital status (married or partnered, unpartnered), and whether or not the respondent was insured. We also controlled for the individuals’ occupational group, classifying individuals who were employed by their 4-category occupational group status (white collar, blue collar, farm and forestry, and service) and those who were not in the labor force (individuals who reported that they were looking for work, or not working at a job or business, and not looking for work). We could not use this 5-category occupational and employment designations in our posthoc statistical analyses as 138 of the 170 Mexican-descent individuals in the farm, or fishing or forestry category were foreign-born, which means there was little variation in this category among Mexican-identified individuals, so we combined this category with the blue-collar category in the posthoc analyses. In addition, we controlled for region of the United States (North, Midwest, South, and West) and whether or not the respondent reported using the internet at least once a day. To minimize collinearity, we did not control for English proficiency, as we found it significantly negatively correlated with being US-born among our sample of US-born whites and Latinos (correlation=−0.66).

### Statistical Analysis

We ran multivariable binary logistic regressions to test the relationship between ethnicity and nativity and internet use. We then used a binary logistic regression to test the relationship between ethnicity and nativity and our 2 HISB and 3 patient portal outcomes among internet users. The sample was weighted according to survey directions. Due to the small number of individuals among some of the Latino ethnic and nativity groups, we do not report groups where too few individuals use the technology in question to draw a statistical conclusion.

## Results

### Demographics

The majority (38,256/49,259, 82.6%; 95% CI 82.1-83.2) of respondents reported using the internet (Table 1), and of 65.05% (25,209/38,256; 95% CI 64.3-65.8) respondents reported looking up health information on the internet; however, less than 13% of respondents reported using a computer to engage in any of the surveyed Web-based patient portal channels (Table 2). Among internet users, Mexican-identified individuals (Mexicans) were the largest US- and foreign-born subgroups (2337/38,256, 6.24%; 95% CI 5.8-6.8 and 1358/38,256, 4.1%; 95% CI 3.8-4.4, respectively). The next largest US-born subgroup was Puerto Ricans (414/38,256, 1.2%; 95% CI 0.97-1.24). There were more foreign-born Central and South Americans (739/38,256) and other Latinos (307/38,256) than US-born individuals within these subgroupings (246/38,256 and 139/38,256, respectively).

**Table 1:** Characteristics of US-born non-Hispanic whites and Latinos (National Health Interview Survey, 2015-16). N=49,259.

Variables			Statistics, n (%)	95% CI
**Dependent variables**				
	Use the internet		38,256 (82.61)	82.05-83.15
**Independent variables**				
	**Ethnicity by nativity NH^a^** **whites and Latino subgroup affiliation**			
		US-born NH White	40,732 (80.36)	79.34-81.34
		US-born Mexican	2585 (6.06)	5.56-6.79
		Foreign-born Mexican	2718 (6.28)	5.8-6.79
		US-born Puerto Rican	489 (1.09)	0.97-1.24
		Foreign-born Puerto Rican	476 (0.99)	086-1.14
		US-born Central or South American	268 (0.71)	0.59-0.85
		Foreign-born Central or South American	1195 (2.86)	2.59-3.16
		US-born other Latino	157 (0.42)	0.33-0.52
		Foreign-born other Latino	631 (1.22)	1.04-1.43
	**Age, years**			
		18-30	8837 (22.21)	21.58-22.85
		31-54	18,386 (40.29)	39.65-40.94
		>55	22,036 (37.5)	36.81-38.19
		**Sex**			
		Female	26,633 (51.18)	50.57-51.79
		Male	22,626 (48.82)	48.21-49.43
	**Education**			
		Below high school	6152 (12.37)	11.87-12.88
		High school or general education development	12,258 (24.92)	24.33-25.52
		Some college	15,633 (31.53)	30.91-32.16
		Bachelor’s or above (reference)	15,072 (31.18)	30.41-31.96
	**Income to federal poverty level (FPL) ratio**			
		Poor (income to FPL ratio 0-0.99)	6266 (10.66)	10.22-11.12
		Near poor (income to FPL ratio 1-1.99)	8843 (17.32)	16.8-17.85
		Above poor (income to FPL ratio >2)	31,788 (72.02)	71.31-72.73
	**Married or partnered**			
		Married or partnered	26,135 (63.48)	62.83-64.13
		Single or widowed	23,040 (36.52)	35.87-37.17
	**Occupational group**			
		White collar	28,234 (58.12)	57.39-58.85
		Service	7615 (15.37)	14.89-15.86
		Farm and forestry	475 (0.86)	0.73-1.01
		Blue Collar	10,168 (21.03)	20.46-21.61
		Not in labor force	2138 (4.63)	4.32-4.96
	**Health insurance**			
		Covered	44,471 (90.16)	89.71-90.6
		Not covered	4613 (9.84)	9.4-10.29

	**Region**			
		Northeast	8473 (17.49)	16.67-18.35
		Midwest	11,801 (24.51)	23.66-25.38
		South	15,617 (34.44)	33.27-35.64
		West	13,368 (23.55)	22.45-24.69

^a^NH: non-Hispanic.

### Internet Use

The percentage of internet users who use each technology varies by ethnicity and nativity (Tables 3-5). A total of 85.6% (32,735/40,732; 95% CI 85.1-86.1) of US-born NH whites report using the internet, whereas 53.76% (1358/2037; 95% CI 51.1-56.4) of US-born Mexicans reported using the internet. Foreign-born Mexicans (633/2037) and foreign-born Central and South Americans (370/739) and foreign-born other Latinos (145/307) had less than 50% of the population report looking up health information online.

We found that US-born Mexicans (OR 0.81, 95% CI 0.66-0.99), foreign-born Mexicans (OR 0.35, 95% CI 0.29-0.42), foreign-born Puerto Ricans (OR 0.62, 95% CI 0.44-0.87), foreign-born Central and South Americans (OR 0.42, 95% CI 0.33-0.53), and foreign-born other Latinos (OR 0.34, 95% CI 0.24-0.49) had lower odds of using the internet than US-born NH whites (Table 6).

### Online Health Information–Seeking Behavior

The relationship between subgroup affiliation and online HISB varied by type of technology. US-born Mexicans (OR 0.77, 95% CI 0.66-0.90), foreign-born Mexicans (OR 0.51, 95% CI 0.43-0.61), foreign-born Central and South Americans (OR 0.53, 95% CI 0.43-0.64), and foreign-born other Latinos (OR 0.56, 95% CI 0.40-0.79) had lower odds of looking up health information online than US-born NH whites (Table 6). Foreign-born Central and South Americans (OR 1.71, 95% CI 1.11-2.62) had higher odds of using a chat group to discuss health information than US-born NH whites, whereas there was no significant difference between all other groups and NH whites regarding the use of chat groups to discuss health information.

### Patient Portal Use

Controlling for age, sex, education, income to FPL, and region, foreign-born Central and South Americans (OR 0.61, 95% CI 0.41-0.92) had lower odds of filling a prescription using a computer than US-born NH whites (Table 7). There was no significant difference using a computer to schedule medical appointments between US-born NH whites and the various Latino ethnic and nativity groups. Foreign-born Mexicans (OR 0.51, 95% CI 0.36-0.72) and foreign-born Central and South Americans (OR 0.51, 95% CI 0.36-0.72) have lower odds of emailing a health care provider than US-born NH whites.

**Table 2:** Characteristics of US-born non-Hispanic whites and Latino internet users (National Health Interview Survey, 2015-16). N=38,256.

Variables			Statistics, n (%)	95% CI
**Dependent variables**				
	Use the internet		—^a^	—
	**Online health information–seeking behavior**			
		Look up health information on the internet	25,209 (65.05)	64.3-65.8
		Use online chat groups to learn about health topics	1634 (4.32)	4.06-4.59
	**Web-based health information technology**			
		Used a computer to fill a prescription	4564 (11.91)	11.43-12.41
		Used a computer to schedule an appointment with a health care provider	4834 (13.43)	12.87-14.01
		Used a computer to communicate with a health care provider by email	5653 (15.2)	14.6-15.82
**Independent variables**				
	**Ethnicity by nativity NH^b^** **whites and Latino subgroup affiliation**			
		US-born NH white	32,735 (83.28)	82.41-84.11
		US-born Mexican	2337 (6.24)	5.75-6.76
		Foreign-born Mexican	1358 (4.08)	3.76-4.44
		US-born Puerto Rican	414 (1.18)	1.03-1.35
		Foreign-born Puerto Rican	276 (0.78)	0.66-0.93
		US-born Central and South American	246 (0.82)	0.68-0.99
		Foreign-born Central and South American	739 (2.33)	2.07-2.63
		US-born other Latino	139 (0.46)	0.37-0.59
		Foreign-born other Latino	307 (0.8)	0.67-0.96
	**Age, years**			
		18-30	8191 (25.37)	24.64-26.12
		31-54	15,772 (42.97)	42.26-43.69
		>55	14,293 (31.66)	30.94-32.39
	**Sex**			
		Female	20,790 (51.51)	50.83-52.19
		Male	17,466 (48.49)	47.81-49.19
	**Education**			
		Below high-school	2488 (7.09)	6.71-70.5
		High-school or general education development	8253 (22.20)	21.59-22.83
		Some college	13,348 (34.26)	33.54-34.98
		Bachelor’s or above (reference)	14,106 (36.45)	35.58-37.32
	**Income to federal poverty level (FPL) ratio**			
		Poor (income to FPL ratio 0-0.99)	3899 (8.41)	7.99-8.86
		Near poor (income to FPL ratio 1-1.99)	5630 (14.34)	13.81-14.88
		Above poor (income to FPL ratio >2)	27,237 (77.25)	76.55-77.93
	**Married or partnered**			
		Married or partnered	21,536 (65.36)	64.63-66.09
		Single or widowed	16,664 (34.64)	33.91-35.37
	**Occupational group**			
		White collar	24,659 (64.45)	63.71-65.18
		Service	5403 (14.28)	13.76-14.81
		Farm and forestry	228 (0.51)	0.42-0.6
		Blue collar	6452 (17.48)	16.94-18.04
		Not in labor force	1028 (3.29)	3.01-3.59
	**Use the internet once a day or more often**			
		Use the internet once a day or more often	28,591 (78.32)	77.41-79.21
		Use the internet less than once a day	7804 (21.68)	20.79-22.59
	**Health insurance**			
		Covered	34,801 (91.21)	90.78-91.62
		Not covered	3319 (8.79)	8.38-9.22
	**Region**			
		Northeast	6576 (17.68)	16.8-18.59
		Midwest	9273 (24.9)	23.97-25.86
		South	11,653 (33.27)	32.07-34.5
		West	10,772 (24.15)	23.02-25.31

^a^—: not applicable.

^b^NH: non-Hispanic.

**Table 3:** Percent of individuals in each ethnic-native group who use the internet and look up health information online (National Health Interview Survey, 2015-16).

Ethnicity by nativity	Population: use the internet		Population: look up health information	
	n (%)	95% CI	n (%)	95% CI
US-born non-Hispanic whites	32,735 (85.6)	85.1-86.1	22,167 (67.3)	66.6-68.1
US-born Mexican	2037 (85.0)	82.7-87.1	1228 (58.2)	55.0-61.4
Foreign-born Mexican	1358 (53.8)	51.1-56.4	633 (45.1)	41.6-48.7
US-born Puerto Rican	414 (88.9)	84.0-92.4	247 (58.2)	52.1-64.0
Foreign-born Puerto Rican	276 (65.2)	60.2-70.0	155 (56.3)	48.2-64.0
US-born Central and South American	246 (95.9)	92.5-97.7	171 (69.1)	60.6-76.5
Foreign-born Central and South American	739 (67.3)	63.6-70.8	370 (48.8)	44.1-53.5
US-born other Latino	139 (91.4)	83.7-95.6	90 (61.7)	51.1-71.3
Foreign-born other Latino	307 (55.4)	50.0-60.7	145 (45.8)	38.8-52.9

**Table 4:** Percent of individuals in each ethnic-native group who are internet users and use a chat group or fill a prescription online (National Health Interview Survey, 2015-16).

Ethnicity by nativity	Population: use chat group		Population: fill prescription	
	n (%)	95% CI	n (%)	95% CI
US-born whites	1349 (4.2)	4.0-4.5	4170 (12.9)	12.3-13.4
US-born Mexican	93 (4.1)	3.1-5.4	184 (9.2)	7.7-11.2
Foreign-born Mexican	73 (4.7)	3.6-6.2	62 (5.0)	3.7-6.7
US-born Puerto Rican	21 (3.4)	2.0-5.8	37 (8.2)	5.0-13.2
Foreign-born Puerto Rican	15 (6.4)	3.2-12.5	19 (6.0)	3.5-9.9
US-born Central and South American	12 (3.5)	1.7-6.9	30 (8.9)	5.5-14.0
Foreign-born Central and South American	52 (8.0)	5.6-11.3	46 (6.5)	4.5-9.2
US-born other Latino	3 (2.1)	0.5-8.4	8 (4.3)	1.9-9.4
Foreign-born other Latino	16 (3.9)	2.0-7.5	7 (2.3)	1.0-5.3

**Table 5:** Percent of individuals in each ethnic-native group who use the internet and look up health information online (National Health Interview Survey 2015-16).

Ethnicity by nativity	Population: schedule medical appointment		Population: email providers	
	n (%)	95% CI	n (%)	95% CI
US-born whites	4278 (14.1)	13.5-14.7	5177 (16.5)	15.9-17.2
US-born Mexican	247 (12.7)	10.8-14.8	209 (10.9)	9.1-13.1
Foreign-born Mexican	89 (6.8)	5.3-8.7	66 (4.8)	3.5-6.5
US-born Puerto Rican	47 (12.2)	8.4-17.2	46 (9.3)	6.3-13.5
Foreign-born Puerto Rican	25 (7.9)	5.0-12.3	17 (6.0)	3.6-9.9
US-born Central and South American	47 (17.1)	11.7-24.3	40 (16.2)	10.5-24.2
Foreign-born Central and South American	71 (9.5)	7.2-12.4	71 (9.4)	7.0-12.5
US-born other Latinos	13 (10.4)	5.8-17.7	11 (5.9)	3.0-11.3
Foreign-born other Latinos	17 (5.3)	3.1-8.9	16 (5.6)	2.9-10.6

**Table 6:** Correlation between (1) Latino subgroup affiliation and internet use and (2) Latino subgroup affiliation and online health information seeking behavior among internet users, controlling for age, sex, education, income to federal poverty level, marital or partnered status, occupation, insurance status, daily internet use, and region using the National Health Interview Survey 2015-16.

Variables^a^	Dependent variable: use internet (n=45,449), OR^b^ (95% CI)	Internet users	
		Dependent variable: look up health information (n=34,467), OR (95% CI)	Dependent variable: use chat group (n=34,484), OR (95% CI)
US-born white	1.00 (Reference)	1.00 (Reference)	1.00 (Reference)
US-born Mexican	0.81 (0.66-0.99)^c^	0.77 (0.66-0.9)^c^	1.02 (0.74-1.42)
US-born Puerto Rican	1.2 (0.78-1.84)	0.75 (0.56-1.01)	0.96 (0.54-1.7)
US-born Central and South American	1.44 (0.75-2.74)	1.16 (0.76-1.77)	0.92 (0.44-1.91)^d^
US-born other Latino	1 (0.41-2.47)	0.83 (0.49-1.4)	0.5 (0.11-2.18)^d^
Foreign-born Mexican	0.35 (0.29-0.42)^c^	0.51 (0.43-0.61)^c^	1.28 (0.9-1.82)
Foreign-born Puerto Rican	0.62 (0.44-0.87)^c^	0.82 (0.57-1.17)	1.27 (0.54-2.99)
Foreign-born Central and South American	0.42 (0.33-0.53)^c^	0.53 (0.43-0.64)^c^	1.71 (1.11-2.62)^c^
Foreign-born other Latino	0.34 (0.24-0.49)^c^	0.56 (0.4-0.79)^c^	0.92 (0.43-1.97)^d^

^a^Control variables not listed include age, sex, education, income to federal poverty level ratio, occupational group, insurance status, daily internet use, and region.

^b^OR: odds ratio.

^c^Indicates values are significant.

^d^Too few individuals use the technology in question to draw a statistical conclusion.

**Table 7:** Correlation between Latino subgroup affiliation and online patient portal use among internet users controlling for age, sex, education, income to federal poverty level, marital or partnered status, occupation, insurance status, daily internet use, and region using the National Health Interview Survey 2015-16. N=46,480.

Variables^a^	Use of health information technology		
	Dependent variable: fill prescription (n=34,483), OR^b^ (95% CI)	Dependent variable: schedule medical appointment (n=34,477), OR (95% CI)	Dependent variable: email provider (n=34,480), OR (95% CI)
US-born non-Hispanic white	1.00 (Reference)	1.00 (Reference)	1.00 (Reference)
US-born Mexican	1.01 (0.81-1.26)	1.01 (0.83-1.23)	0.85 (0.68-1.06)
US-born Puerto Rican	0.94 (0.53-1.68)	1.12 (0.71-1.77)	0.8 (0.5-1.27)
US-born Central and South American	1.15 (0.67-2)	1.21 (0.75-1.96)	1.1 (0.63-1.9)
US-born other Latino	0.48 (0.2-1.15)^c^	0.84 (0.44-1.59)^c^	0.43 (0.2-0.93)^c^
Foreign-born Mexican	0.72 (0.51-1.01)	0.86 (0.64-1.15)	0.51 (0.36-0.72)^d^
Foreign-born Puerto Rican	0.74 (0.41-1.34)	0.86 (0.51-1.46)	0.57 (0.32-1)^c^
Foreign-born Central and South American	0.61 (0.41-0.92)^d^	0.82 (0.59-1.13)	0.7 (0.5-0.99)^d^
Foreign-born other Latino	0.26 (0.1-0.68)^c^	0.6 (0.32-1.12)^c^	0.57 (0.27-1.17)^c^

^a^Control variables not listed include age, sex, education, income to federal poverty level ratio, occupational group, insurance status, daily internet use, and region.

^b^OR: odds ratio.

^c^Too few individuals use the technology in question to draw a statistical conclusion.

^d^Indicates values are significant.

### Posthoc Analysis

We chose to analyze Mexican-identified individuals because they are the largest Hispanic ethnic subgroup in the United States [34] and contain enough individuals to do a subgroup analysis of a single Latino subethnic group (N=5310). As age is thought to be a factor in the use of social media and other Web-based communication channels [35], we conducted 2 posthoc analyses of Mexican-identified individuals to see if age was significant in predicting internet use, online HISB, or the use of HIT. We also conducted a third posthoc analysis to see if nativity and insurance status were significant predictors of online HISB and patient portal use among Mexican-identified individuals who used the internet. The supplementary file for posthoc analyses is provided in Multimedia Appendix 1.

#### Examining Ages 18 to 30 Years

We first examined Mexican-identified individuals to see if individuals aged 18 to 30 years behaved differently than individuals aged 31 years or older (posthoc analysis tables available from author on request). We found that those aged 18 to 30 year had higher odds of using the internet (OR 3.46, 95% CI 2.61-4.59). Among individuals who used the internet, individuals aged 18 to 30 years had lower odds of looking up health information online (OR 0.75, 95% CI 0.58-0.96), filling prescriptions online (OR 0.45, 95% CI 0.27-0.75), and using a chat group to learn about health topics (OR 0.55, 95% CI 0.32-0.96) than individuals aged 31 or older. There was no significant difference between these 2 age groups with regard to emailing a health care provider (OR 0.64, 95% CI 0.4-1.02).

#### Examining Ages 55 Plus

We then examined Mexicans who used the internet to see if older individuals (aged 55 years or older) were significantly different than other age groups. Individuals who were 55 years or older had lower odds of using the internet (OR 0.13, 95% CI 0.1-0.17) and higher odds of filling prescriptions online (OR 1.91, 95% CI 1.17-3.14). There was no significant difference between individuals who were older than 55 years and individuals aged 18 to 54 years with respect to looking up health information online (OR 0.94, 95% CI 0.71-1.23), scheduling a medical appointment online (OR 1.25, 95% CI 0.77-2.01), emailing a health care provider (OR 1.66, 95% CI 0.97-2.85), or using a chat group to learn about health topics (OR 2.02, 95% CI 0.97-4.18).

#### Within-Group Nativity Differences

We then examined online HISB among Mexican-identified individuals who used the internet to understand differences in online HISB and patient portal use by nativity. We found that US-born individuals had higher odds (OR 52.9, 95% CI 1.2-1.93) of looking up health information online compared with foreign-born individuals. There was no significant difference between US- and foreign-born Mexicans regarding the use of chat groups to learn about a health topic. US-born Mexican-identified individuals had significantly higher odds of using a computer to fill a prescription (OR 1.58, 95% CI 1.02-2.44) or emailing a provider (OR 1.73, 95% CI 1.13-2.64) than foreign-born Mexican-identified individuals. There was no significant difference between US- and foreign-born Mexicans who used the internet with regard to scheduling a medical appointment online. When we examined the relationship between having insurance and HISB or patient portal use among all Mexican-identified individuals who used the internet, we found that individuals who were insured had higher odds of filling prescriptions online (OR 2.66, 95% CI 1.35-5.25), making medical appointments online (OR 2.08, 95% CI 1.25-3.47), or using email to contact their provider (OR 2.45, 95% CI 1.35-4.46).

## Discussion

### Principal Findings

Our findings show that although the use of Web-based patient portals is growing [1], there are significant disparities in online HISB and patient portal use in the United States between NH whites and various Latino subgroups after controlling for individual-level differences; however, these disparities vary by subgroup affiliation, nativity, and mode of access. Our findings also imply a gap in access between NH whites and the Mexican subpopulation as a whole, whereas foreign-born groups experience similar disparities across ethnic groups. Although recent reports have shown that internet access among foreign-born Latinos is increasing [17,33], our data show that the Mexican population and foreign-born Latinos in general should be a target for increased access.

Our findings regarding online HISB and patient portals are in line with previous literature showing low internet efficacy among Latinos as compared with whites [8,9,13,36] and showing that Latinos may be less likely to use patient portals [4,37]. However, we expand upon previous research by showing that mode of access, nativity, and subgroup affiliation matter. Not all internet channels are equally likely to reach subpopulations within the Latino population. The need to pay attention to nativity and subgroup or regional affiliation among Latinos is particularly important in light of other studies that show that the internet is becoming the most common source of health information for all ethnic groups [38]. In addition, although emerging research shows that Spanish speakers view Web-based health portals positively [39] and computer-assisted interventions are being deployed more frequently among US Latinos [40], our research implies that the deployment of such interventions online will have uneven penetration among Latinos, particularly foreign-born individuals and Mexican-identified persons. Our findings also support previous research showing that certain portions of the US Mexican-identified population, such as farmworkers, do not seek health information online [36].

Generally, except for US-born Central and South Americans, our respondents are not looking up health information at the same rate as NH whites, although this result is only statistically significant for some groups. The pattern of the direction of the findings for using chat groups is the same for all groups except US-born Puerto Ricans, US-born Central and South Americans, and US- and foreign-born other Latinos. It is only significant for foreign-born Central and South Americans such that they are more likely than NH whites. It may be that this group uses chat groups that allow them to maintain relationships with social networks at home, perhaps allowing people to have contact with others from a similar cultural background. Our data suggest a trend where foreign-born Latinos use patient portals less often than US-born NH whites; however, the difference is only significant for some groups. There are a number of different possible reasons for this. Latinos who are less comfortable with English trust mediated sources less [41]. Previous literature has shown that Latinos may rely on and prefer interpersonal channels [42,43], and 1 study suggested that although some Latinos view patient portals positively, they are less interested in accessing their own medical records online [44], whereas an analysis of focus groups found that Latinos preferred portals that were easy to use and accessible on a mobile phone [43]. Our findings suggest that medical care providers could encourage the use of portals by their patients from these subpopulations by providing handouts, short conversations during appointments, or other aids to encourage use. Alternative forms of patient portals, including portals that are in Spanish, culturally tailored, encourage more personal forms of patient-provider interactions and are easily accessible on mobile phones, which may be a more effective way to reach this target population.

### Mexican-Identified Subpopulation

Our posthoc analysis of the Mexican-identified subpopulation shows that nativity also matters within Latinos groups, which is in line with other studies that use Spanish as a measure of acculturation [5,36] and with previous state-level studies showing differences between US- and foreign-born Latinos [9]. US-born individuals within the Mexican subethnicity were generally more likely to use HISB or patient portals than their foreign-born counterparts (although this was not true across the board), which is in line with other studies that show that more acculturated Mexican Americans are willing to use patient portals [44]. Among Mexican-identified persons, individuals who were insured had higher odds of using patient portals more within this group, which may indicate that access to health care is an important factor for patient portal use among Mexican Americans.

### Age Effects

Previous studies have shown that individuals who are younger than 35 years are less likely to use patient portals [4], and our posthoc analysis found that younger Mexicans had lower odds of looking up health information online, filling prescriptions online, scheduling a medical appointment online, and using a chat group to learn about health topics, which may be because of the fact that younger individuals have less health problems than older individuals or that individuals with multiple chronic conditions are more likely to use patient portals than those without chronic conditions [45,46]. Although some studies have shown that older Latino adults were less likely to have activated patient portals as compared with NH whites [16], we found that among Mexican-identified individuals, people aged 55 years or older had higher odds of filling prescriptions online, which may be related to their health status. Future research should examine which Web-based apps, programs, and services are being used, especially by foreign-born Latinos and the Mexican-identified population. Such research will allow effective dissemination of health information to foreign-born individuals and potentially reach individuals who are not using other avenues to obtain health information.

### Limitations

As this is a secondary data analysis of information, the primary limitation is the use of cross-sectional data, which means there is no causal analysis possible. The data are self-reported; therefore, recall bias and other factors may affect measurement. Furthermore, literacy significantly influences online behavior [47-49], and no measures of online literacy, including health or computer literacy, are assessed in the NHIS. For some groups, too few people reported engaging in HIT to report the findings as statistically significant; consequently, in 1 of our posthoc analyses, we combined US- and foreign-born individuals. Furthermore, as the questions on internet use, HISB, and HIT were asked to all respondents, it was possible that the respondents’ answers were inconsistent, where they could answer that they did not use the internet but report that they emailed their provider.

### Conclusions

Our findings indicate that the significant gaps in online health-related behaviors between NH whites and Latinos vary by subgroup affiliation, nativity, and mode of access. Although foreign-born Latinos of all ethnic subgroups have lower rates of online HISB, US-born Mexicans also experience lower odds than US-born NH whites. This is important in terms of information dissemination because it suggests that many of the channels that are increasingly being used by NH whites are not effective for communicating with many Latino groups. In addition, our results highlight the need for additional training in internet and patient portal technologies as health information becomes increasingly available online for many of the groups in this study. Without additional training and given that there is such a variation in health behaviors and outcomes, care should be taken when deciding which online channels will be used to reach these populations.

## Appendix

Multimedia Appendix 1 Supplementary file for posthoc analyses.

## Abbreviations

FPL: federal poverty level
HISB: health information–seeking behavior
HIT: health information technology
NH: non-Hispanic
NHIS: National Health Interview Survey
OR: odds ratio

## References

[1] Cohen RA, Stussman B Centers for Disease Control and Prevention.

[2] McCann E Healthcare IT News.

[3] Kruse CS, Argueta DA, Lopez L, Nair A (2015). Patient and provider attitudes toward the use of patient portals for the management of chronic disease: a systematic review. J Med Internet Res.

[4] Irizarry T, DeVito DA, Curran CR (2015). Patient portals and patient engagement: a state of the science review. J Med Internet Res.

[5] De Jesus M, Xiao C (2012). Predicting internet use as a source of health information: a “Language Divide” among the Hispanic population in the United States. Policy Internet.

[6] Choi NG, Dinitto DM (2013). The digital divide among low-income homebound older adults: internet use patterns, eHealth literacy, and attitudes toward computer/internet use. J Med Internet Res.

[7] Cunningham PJ, Hibbard J, Gibbons CB (2011). Raising low 'patient activation' rates among Hispanic immigrants may equal expanded coverage in reducing access disparities. Health Aff (Millwood).

[8] Brown A, Lopez G, Lopez M (2016). Pew Research Center.

[9] Gonzalez M, Sanders-Jackson A, Emory J (2016). Online health information-seeking behavior and confidence in filling out online forms among Latinos: a cross-sectional analysis of the California Health Interview Survey, 2011-2012. J Med Internet Res.

[10] Chaet AV, Morshedi B, Wells KJ, Barnes LE, Valdez R (2016). Spanish-language consumer health information technology interventions: a systematic review. J Med Internet Res.

[11] Christopher Gibbons M (2011). Use of health information technology among racial and ethnic underserved communities. Perspect Health Inf Manag.

[12] Perrin A Pew Research Center.

[13] López L, Grant RW (2012). Closing the gap: eliminating health care disparities among Latinos with diabetes using health information technology tools and patient navigators. J Diabetes Sci Technol.

[14] Peña-Purcell N (2008). Hispanics' use of internet health information: an exploratory study. J Med Libr Assoc.

[15] Goldzweig CL, Orshansky G, Paige NM, Towfigh AA, Haggstrom DA, Miake-Lye I, Beroes JM, Shekelle PG (2013). Electronic patient portals: evidence on health outcomes, satisfaction, efficiency, and attitudes: a systematic review. Ann Intern Med.

[16] Gordon NP, Hornbrook MC (2016). Differences in access to and preferences for using patient portals and other ehealth technologies based on race, ethnicity, and age: a database and survey study of seniors in a large health plan. J Med Internet Res.

[17] Sarkar U, Karter AJ, Liu JY, Adler NE, Nguyen R, López A, Schillinger D (2011). Social disparities in internet patient portal use in diabetes: evidence that the digital divide extends beyond access. J Am Med Inform Assoc.

[18] Wallace LS, Angier H, Huguet N, Gaudino JA, Krist A, Dearing M, Killerby M, Marino M, DeVoe JE (2016). Patterns of electronic portal use among vulnerable patients in a nationwide practice-based research network: From the OCHIN Practice-Based Research Network (PBRN). J Am Board Fam Med.

[19] Finney Rutten LJ, Hesse BW, Moser RP, Ortiz Martinez AP, Kornfeld J, Vanderpool RC, Byrne M, Tortolero LG (2012). Socioeconomic and geographic disparities in health information seeking and internet use in Puerto Rico. J Med Internet Res.

[20] Lee YJ, Boden-Albala B, Larson E, Wilcox A, Bakken S (2014). Online health information seeking behaviors of Hispanics in New York City: a community-based cross-sectional study. J Med Internet Res.

[21] Lee YJ, Boden-Albala B, Quarles L, Wilcox A, Bakken S (2012). Predictors of health information-seeking behaviors in Hispanics. NI 2012 (2012).

[22] Selsky C, Luta G, Noone A, Huerta EE, Mandelblatt JS (2013). Internet access and online cancer information seeking among Latino immigrants from safety net clinics. J Health Commun.

[23] Stepler R, Lopez M Pew Research Center.

[24] Patten E Pew Research Center.

[25] Aponte J (2009). Diabetes-related risk factors across Hispanic subgroups in the Hispanic health and nutritional examination survey (1982-1984). Public Health Nurs.

[26] Oquendo MA, Lizardi D, Greenwald S, Weissman MM, Mann JJ (2004). Rates of lifetime suicide attempt and rates of lifetime major depression in different ethnic groups in the United States. Acta Psychiatr Scand.

[27] Kerner JF, Breen N, Tefft MC, Silsby J (1998). Tobacco use among multi-ethnic Latino populations. Ethn Dis.

[28] Fox RS, Mills SD, Roesch SC, Sotres-Alvarez D, Gonzalez P, Bekteshi V, Cai J, Lounsbury DW, Talavera GA, Penedo FJ, Malcarne VL (2018). Perceptions of cancer risk/efficacy and cancer-related risk behaviors: results from the HCHS/SOL Sociocultural Ancillary Study. Health Educ Behav.

[29] Fox S, Livingston G (2018). Pew Research Center.

[30] Center for Disease Control and Prevention Centers for Disease Control and Prevention.

[31] (2013). Pew Research Center.

[32] (2013). Pew Research Center.

[33] Brown A, Lopez G, Lopez M (2016). Pew Research Center.

[34] Flores A (2017). Pew Research Center.

[35] (2017). Pew Research Center.

[36] Ginossar T (2016). Predictors of online cancer prevention information seeking among patients and caregivers across the digital divide: a cross-sectional, correlational study. JMIR Cancer.

[37] Ancker JS, Hafeez B, Kaushal R (2016). Socioeconomic disparities in adoption of personal health records over time. Am J Manag Care.

[38] Nguyen AB, Robinson J, O'Brien EK, Zhao X (2017). Racial and ethnic differences in tobacco information seeking and information sources: findings from the 2015 Health Information National Trends Survey. J Health Commun.

[39] Ochoa A, Kitayama K, Uijtdehaage S, Vermillion M, Eaton M, Carpio F, Serota M, Hochman ME (2017). Patient and provider perspectives on the potential value and use of a bilingual online patient portal in a Spanish-speaking safety-net population. J Am Med Inform Assoc.

[40] Klein CH, Kuhn T, Altamirano M, Lomonaco C (2017). C-SAFE: a computer-delivered sexual health promotion program for Latinas. Health Promot Pract.

[41] Clayman ML, Manganello JA, Viswanath K, Hesse BW, Arora NK (2010). Providing health messages to Hispanics/Latinos: understanding the importance of language, trust in health information sources, and media use. J Health Commun.

[42] Elder JP, Ayala GX, Parra-Medina D, Talavera GA (2009). Health communication in the Latino community: issues and approaches. Annu Rev Public Health.

[43] Lyles CR, Allen JY, Poole D, Tieu L, Kanter MH, Garrido T (2016). "I Want to Keep the Personal Relationship With My Doctor": understanding barriers to portal use among African Americans and Latinos. J Med Internet Res.

[44] Trubitt M, Alozie O, Shokar G, Flores S, Shokar NK (2018). Patterns and correlates of internet use, cell phone use, and attitudes toward patient portals among a predominantly Mexican-American clinic population. Telemed J E Health.

[45] Greenberg AJ, Falisi AL, Finney Rutten LJ, Chou WS, Patel V, Moser RP, Hesse BW (2017). Access to electronic personal health records among patients with multiple chronic conditions: a secondary data analysis. J Med Internet Res.

[46] Hopman WM, Harrison MB, Coo H, Friedberg E, Buchanan M, VanDenKerkhof EG (2009). Associations between chronic disease, age and physical and mental health status. Chronic Dis Can.

[47] Cline RJ, Haynes KM (2001). Consumer health information seeking on the internet: the state of the art. Health Educ Res.

[48] Dincer S (2012). A study of the relationship between pupils and parents' computer literacy level and use. Procedia Social Behav Sci.

[49] Coughlin SS, Stewart JL, Young L, Heboyan V, De Leo G (2018). Health literacy and patient web portals. Int J Med Inform.

