# Supplementary material for: Web-Based Health Information Technology: Access Among Latinos Varies by Subgroup Affiliation
Source: J Med Internet Res. 2019 Apr 16;21(4):e10389. doi: 10.2196/10389 (PMC6488958; doi:10.2196/10389)
Supplement: Multimedia Appendix 1 [file jmir_v21i4e10389_app1.pdf]

Supplemental Table 1. Characteristics of Mexican-identified individuals in US (NHIS, 2015-16)

	N	% (95% CI)
**Dependent Variables**		
Use the internet	3,400	69.15% (67.24, 70.99)
online Health Information Seeking Behavior		
Look up health information on the Internet	1,864	36.21% (34.23, 38.24)
Use online chat groups to learn about health topics	166	2.97% (2.43, 3.61)
online Health Information Technology		
Used a computer to fill a prescription	247	5.18% (4.41, 6.08)
Used a computer to schedule an appointment with a health care provider	336	7.05% (6.12, 8.12)
Used a computer to communicate with a health care provider by email	275	5.80% (4.96, 6.78)
**Independent Variables**		
Nativity		
US-born	2,585	49.10% (46.91, 51.30)
Age		
18-30	1,497	32.33% (30.59, 34.12)
31-54	2,607	49.09% (47.17, 51.02)
55 plus	1,206	18.58% (17.09, 20.16)
Sex		
Female	2,953	50.47% (48.63, 52.30)
Male	2,357	49.53% (48.63, 52.37)
Education		
Below High-school	1,989	35.74% (33.79,37.74)
High-school/GED	1,416	27.70% (25.92, 29.55)
Some College	1,276	25.71% (24.00, 27.49)
Bachelor's or above	593	10.86% (9.69, 12.14)
Occupation		
White Collar	1,774	33.83% (31.94, 35.77)
Service Worker	1,334	25.57% (21.91, 25.32)
Blue Collar or Farm/Forestry Worker	1,596	32.16% (30.16, 34.22)
Not in the Labor Force	549	10.44% (9.06, 12.00)
Insurance Status		
Insured	3,799	72.55% (70.56, 74.46)
Income to federal poverty level ratio		
Poor (income to federal poverty level ratio 0-0.99)	1,316	20.86% (19.32, 22.50)
Near Poor (income to federal poverty level ratio 1-1.99)	1,537	30.27% (28.51, 32.10)
Above Poor (income to federal poverty level ratio >2.00)	2,208	48.86% (46.77, 50.96)
Married or partnered		
Married/Partnered	2,941	61.90% (60.01, 63.76)
Single/Widowed	2,359	38.10% (36.24, 39.99)
Region		
Northeast	111	2.31% (1.68, 3.15)
Midwest	613	11.58% (9.87, 13.55)

South	1,715	33.96% (30.15, 38.00)
West	2,871	52.15% (48.24, 56.03)
N	5,211	

Supplemental Table 2. Correlation between being age 18-30 and internet use, and online health information seeking behavior (HISB) and Health Information Technology use controlling for age, sex, education, income to federal poverty level, marital/partnered status, and region among Mexican adults living in the US using the NHIS 2015-16 (n=4,881)

		internet users				
	use internet	look up health information	use chat group	fill prescription	schedule medical appointment	email provider
	OR (95% CI)	OR (95% CI)	OR (95% CI)	OR (95% CI)	OR (95% CI)	OR (95% CI)
US-born	**1.87 (1.44, 2.42)**	**1.51 (1.19, 1.91)**	0.87 (0.53, 1.44)	**1.61 (1.04, 2.5)**	1.31 (0.93, 1.86)	**1.76 (1.15, 2.71)**
Age 18 to 30	**3.46 (2.61, 4.59)**	**0.75 (0.58, 0.96)**	**0.55 (0.32, 0.96)**	**0.45 (0.27, 0.75)**	0.76 (0.51, 1.13)	0.64 (0.4, 1.02)
Female	**0.72 (0.57, 0.91)**	**1.64 (1.31, 2.06)**	1.29 (0.79, 2.09)	1.47 (0.95, 2.3)	1.46 (1, 2.15)	**2.16 (1.42, 3.29)**
Below High-school	**0.07 (0.04, 0.14)**	**0.25 (0.17, 0.36)**	0.59 (0.3, 1.18)	**0.29 (0.13, 0.63)**	**0.28 (0.13, 0.61)**	**0.22 (0.1, 0.49)**
High-school/GED	**0.19 (0.1, 0.35)**	**0.37 (0.26, 0.53)**	0.82 (0.43, 1.55)	**0.29 (0.16, 0.51)**	**0.52 (0.33, 0.83)**	**0.28 (0.17, 0.48)**
Some College	**0.38 (0.2, 0.74)**	**0.6 (0.43, 0.83)**	1.04 (0.59, 1.82)	**0.41 (0.26, 0.66)**	**0.58 (0.37, 0.9)**	**0.51 (0.33, 0.78)**
Poor	**0.47 (0.36, 0.61)**	1.2 (0.9, 1.59)	**1.61 (1.01, 2.55)**	0.81 (0.43, 1.55)	0.67 (0.39, 1.17)	0.51 (0.24, 1.06)
Near Poor	**0.7 (0.54, 0.91)**	1.02 (0.78, 1.34)	1.14 (0.65, 2)	0.61 (0.36, 1.06)	**0.55 (0.36, 0.85)**	0.76 (0.47, 1.23)
Married/Partnered	**1.26 (1.01, 1.56)**	1.02 (0.82, 1.26)	1.11 (0.68, 1.8)	**1.63 (1.08, 2.45)**	**1.42 (1, 2.02)**	1.36 (0.92, 2.01)
Service Worker	**0.53 (0.39, 0.71)**	0.95 (0.73, 1.23)	0.99 (0.57, 1.73)	0.99 (0.56, 1.73)	1.09 (0.68, 1.75)	1.2 (0.71, 2.02)
Blue Collar or Farm/Forestry Worker	**0.33 (0.24, 0.45)**	**0.69 (0.52, 0.92)**	0.84 (0.41, 1.73)	0.86 (0.49, 1.53)	**0.53 (0.31, 0.93)**	0.69 (0.37, 1.29)
Not in the Labor Force	**0.3 (0.21, 0.44)**	0.83 (0.52, 1.31)	0.79 (0.31, 2.03)	0.96 (0.36, 2.58)	0.65 (0.32, 1.32)	**0.38 (0.15, 0.92)**
Insured	**0.74 (0.59, 0.92)**	0.98 (0.74, 1.29)	0.86 (0.53, 1.39)	**2.79 (1.42, 5.46)**	**2.1 (1.26, 3.51)**	**2.54 (1.4, 4.62)**
Northeast	0.85 (0.43, 1.65)	1.05 (0.51, 2.15)	0.95 (0.26, 3.55)	1.3 (0.41, 4.17)	1.93 (0.8, 4.66)	1 (0.33, 3.09)
Midwest	0.95 (0.68, 1.33)	1.03 (0.75, 1.42)	0.61 (0.3, 1.27)	0.83 (0.5, 1.39)	0.74 (0.45, 1.19)	0.76 (0.38, 1.51)
South	**0.67 (0.53, 0.85)**	1.01 (0.8, 1.27)	0.91 (0.58, 1.45)	**0.62 (0.42, 0.92)**	0.74 (0.5, 1.09)	**0.47 (0.31, 0.69)**
Uses the Internet Once a Day or More Often		1.28 (0.98, 1.67)	0.88 (0.55, 1.41)	1.3 (0.8, 2.1)	1.23 (0.81, 1.87)	1.2 (0.69, 2.08)
Constant	**33.43 (16.54, 67.58)**	1.46 (0.88, 2.42)	**0.08 (0.03, 0.19)**	**0.05 (0.02, 0.14)**	**0.08 (0.03, 0.2)**	**0.05 (0.02, 0.13)**

Note – bolded items are significant

Supplemental Table 3. Correlation between being age 55 or older and internet use, and online health information seeking behavior (HISB) and patient portal use controlling for age, sex, education, income to federal poverty level, marital/partnered status, and region among Mexican adults living in the US using the NHIS 2015-16 (n=4,881)

		internet users				
	use internet	look up health information	use chat group	fill prescription	schedule medical appointment	email provider
	OR (95% CI)	OR (95% CI)	OR (95% CI)	OR (95% CI)	OR (95% CI)	OR (95% CI)
US-born	**2.46 (1.89, 3.21)**	**1.43 (1.13, 1.81)**	0.79 (0.48, 1.27)	1.42 (0.93, 2.17)	1.25 (0.9, 1.75)	**1.62 (1.07, 2.46)**
Age 55 plus	**0.13 (0.1, 0.17)**	0.94 (0.71, 1.23)	2.02 (0.97, 4.18)	**1.91 (1.17, 3.14)**	1.25 (0.77, 2.01)	1.66 (0.97, 2.85)
Female	**0.71 (0.56, 0.9)**	**1.65 (1.31, 2.06)**	1.31 (0.81, 2.13)	1.44 (0.94, 2.22)	1.46 (1, 2.13)	**2.14 (1.42, 3.23)**
Below High-school	**0.07 (0.04, 0.13)**	**0.25 (0.17, 0.36)**	0.61 (0.31, 1.22)	**0.3 (0.13, 0.66)**	**0.28 (0.13, 0.62)**	**0.22 (0.1, 0.51)**
High-school/GED	**0.18 (0.1, 0.34)**	**0.36 (0.25, 0.51)**	0.8 (0.42, 1.52)	**0.28 (0.16, 0.49)**	**0.51 (0.32, 0.81)**	**0.27 (0.16, 0.46)**
Some College	**0.39 (0.2, 0.77)**	**0.58 (0.42, 0.8)**	1.02 (0.57, 1.8)	**0.41 (0.25, 0.65)**	**0.58 (0.37, 0.89)**	**0.5 (0.32, 0.77)**
Poor	**0.41 (0.31, 0.53)**	1.16 (0.87, 1.55)	**1.63 (1.02, 2.6)**	0.81 (0.42, 1.55)	0.67 (0.39, 1.16)	0.51 (0.25, 1.07)
Near Poor	**0.63 (0.48, 0.82)**	1.01 (0.76, 1.32)	1.16 (0.66, 2.06)	0.61 (0.35, 1.05)	**0.55 (0.36, 0.85)**	0.76 (0.47, 1.22)
Married/Partnered	0.96 (0.77, 1.21)	1.12 (0.91, 1.38)	1.29 (0.82, 2.03)	**1.99 (1.29, 3.07)**	**1.54 (1.08, 2.18)**	**1.52 (1, 2.3)**
Service Worker	**0.53 (0.39, 0.72)**	0.94 (0.72, 1.22)	0.98 (0.56, 1.7)	0.94 (0.54, 1.63)	1.07 (0.67, 1.71)	1.16 (0.69, 1.96)
Blue Collar or Farm/Forestry Worker	**0.33 (0.24, 0.45)**	0.69 (0.52, 0.92)	0.84 (0.42, 1.7)	0.84 (0.47, 1.49)	**0.53 (0.31, 0.92)**	0.67 (0.36, 1.27)
Not in the Labor Force	**0.36 (0.24, 0.53)**	0.78 (0.5, 1.22)	**0.72 (0.28, 1.85)**	0.82 (0.31, 2.12)	0.62 (0.3, 1.25)	**0.35 (0.14, 0.85)**
Insured	0.98 (0.78, 1.24)	1 (0.76, 1.32)	0.82 (0.51, 1.32)	**2.68 (1.36, 5.29)**	**2.09 (1.25, 3.48)**	**2.46 (1.35, 4.48)**
Northeast	0.75 (0.39, 1.43)	1.03 (0.5, 2.14)	0.98 (0.27, 3.57)	1.28 (0.43, 3.8)	1.93 (0.8, 4.67)	1.02 (0.32, 3.2)
Midwest	0.98 (0.68, 1.4)	1.02 (0.74, 1.41)	0.62 (0.3, 1.27)	0.84 (0.5, 1.41)	0.74 (0.45, 1.2)	0.77 (0.39, 1.53)
South	**0.7 (0.55, 0.88)**	1.02 (0.81, 1.29)	0.93 (0.59, 1.47)	**0.63 (0.42, 0.94)**	0.75 (0.51, 1.1)	**0.47 (0.32, 0.7)**
Uses the Internet Once a Day or More Often		1.25 (0.96, 1.63)	0.89 (0.55, 1.43)	1.3 (0.79, 2.12)	1.23 (0.81, 1.88)	1.22 (0.7, 2.14)
Constant	**68.04 (32.47, 142.59)**	1.32 (0.8, 2.17)	**0.06 (0.02, 0.16)**	**0.03 (0.01, 0.1)**	**0.07 (0.03, 0.18)**	**0.04 (0.02, 0.11)**

Note – bolded items are significant

Supplemental Table 4. Correlation between nativity, insurance status, and internet use, and online health information seeking behavior (HISB) and patient portal use use controlling for age, sex, education, income to federal poverty level, marital/partnered status, and region among Mexican adults living in the US using the NHIS 2015-16 (n=4,881)

		internet users				
	use internet	look up health information	use chat group	fill prescription	schedule medical appointment	email provider
	OR (95% CI)	OR (95% CI)	OR (95% CI)	OR (95% CI)	OR (95% CI)	OR (95% CI)
US-born	**2.19 (1.66, 2.89)**	**1.52 (1.2, 1.93)**	0.85 (0.53, 1.37)	**1.58 (1.02, 2.44)**	1.31 (0.92, 1.85)	**1.73 (1.13, 2.64)**
Age 18-30	**12.43 (8.84, 17.48)**	0.88 (0.63, 1.22)	**0.36 (0.16, 0.82)**	**0.32 (0.17, 0.63)**	0.69 (0.39, 1.2)	**0.48 (0.26, 0.89)**
Age 31-54	**6.17 (4.78, 7.95)**	1.22 (0.92, 1.62)	0.59 (0.28, 1.24)	0.65 (0.4, 1.07)	0.88 (0.54, 1.43)	0.69 (0.39, 1.22)
Female	**0.74 (0.59, 0.94)**	**1.64 (1.31, 2.06)**	1.3 (0.8, 2.11)	1.49 (0.95, 2.32)	**1.47 (1, 2.15)**	**2.17 (1.43, 3.31)**
Below High-school	**0.07 (0.04, 0.14)**	**0.25 (0.17, 0.36)**	0.61 (0.3, 1.21)	**0.29 (0.13, 0.64)**	**0.28 (0.13, 0.61)**	**0.22 (0.1, 0.49)**
High-school/GED	**0.18 (0.09, 0.33)**	**0.37 (0.26, 0.52)**	0.83 (0.43, 1.57)	**0.28 (0.16, 0.51)**	**0.52 (0.33, 0.83)**	**0.28 (0.16, 0.47)**
Some College	**0.38 (0.19, 0.74)**	**0.59 (0.43, 0.82)**	1.05 (0.59, 1.86)	**0.41 (0.26, 0.66)**	**0.58 (0.37, 0.91)**	**0.51 (0.33, 0.78)**
Poor	**0.4 (0.3, 0.52)**	1.18 (0.89, 1.58)	**1.67 (1.04, 2.68)**	0.84 (0.44, 1.62)	0.68 (0.39, 1.18)	0.52 (0.25, 1.09)
Near Poor	**0.64 (0.48, 0.83)**	1.01 (0.77, 1.33)	1.18 (0.66, 2.1)	0.63 (0.36, 1.09)	**0.56 (0.36, 0.86)**	0.78 (0.48, 1.26)
Married/Partnered	1.11 (0.89, 1.39)	1.01 (0.82, 1.26)	1.13 (0.69, 1.87)	**1.64 (1.09, 2.49)**	**1.42 (1, 2.02)**	1.37 (0.93, 2.04)
Service Worker	**0.54 (0.4, 0.73)**	0.95 (0.73, 1.23)	0.99 (0.57, 1.72)	0.98 (0.56, 1.72)	1.09 (0.68, 1.74)	1.19 (0.71, 2.02)
Blue Collar or Farm/Forestry Worker	**0.33 (0.24, 0.45)**	**0.69 (0.52, 0.92)**	0.84 (0.41, 1.71)	0.86 (0.48, 1.54)	**0.53 (0.31, 0.93)**	0.68 (0.36, 1.29)
Not in the Labor Force	**0.32 (0.21, 0.48)**	0.83 (0.52, 1.31)	0.78 (0.31, 2)	0.95 (0.36, 2.52)	0.65 (0.32, 1.32)	**0.38 (0.15, 0.92)**
Insured	1 (0.8, 1.26)	0.99 (0.75, 1.31)	0.82 (0.5, 1.32)	**2.66 (1.35, 5.25)**	**2.08 (1.25, 3.47)**	**2.45 (1.35, 4.46)**
Northeast	0.74 (0.39, 1.42)	1.04 (0.51, 2.13)	0.99 (0.27, 3.66)	1.35 (0.43, 4.25)	1.95 (0.81, 4.7)	1.04 (0.34, 3.21)
Midwest	0.96 (0.66, 1.39)	1.03 (0.74, 1.41)	0.62 (0.3, 1.28)	0.84 (0.51, 1.41)	0.74 (0.46, 1.2)	0.77 (0.38, 1.53)
South	**0.69 (0.54, 0.88)**	1.01 (0.8, 1.27)	0.92 (0.58, 1.45)	**0.62 (0.42, 0.92)**	0.74 (0.5, 1.09)	**0.47 (0.32, 0.69)**
Uses the Internet Once a Day or More Often		1.27 (0.98, 1.66)	0.91 (0.56, 1.47)	1.35 (0.82, 2.21)	1.25 (0.82, 1.89)	1.24 (0.7, 2.18)
Constant	**8.33 (3.96, 17.53)**	1.24 (0.73, 2.12)	**0.11 (0.05, 0.27)**	**0.07 (0.02, 0.19)**	**0.09 (0.04, 0.22)**	**0.07 (0.03, 0.18)**

Note – bolded items are significant
